# Supplementary material for: Intestinal Dominance by Multidrug-Resistant Bacteria in Pediatric Liver Transplant Patients
Source: Microbiol Spectr. 2022 Nov 8;10(6):e02842-22. doi: 10.1128/spectrum.02842-22 (PMC9769714; doi:10.1128/spectrum.02842-22)
Supplement: Supplemental file 1 — Supplemental material. Download spectrum.02842-22-s0001.pdf, PDF file, 0.3 MB [file spectrum.02842-22-s0001.pdf]

Supplementary Table 1. Detection of of *bla*$_{CTX-M-1-Family}$, *bla*$_{OXA-1}$, *bla*$_{OXA-48}$, and *bla*$_{VIM}$ in relation to whether or not the patients received carbapenems, non-carbapenem β-lactams, trimethoprim/sulfamethoxazole, and/or aminoglycosides within 30 days before testing the rectal swab for these genes. Three samples were not included in this analysis since the antibiotic consumption data was not available in the information system database.

		Carbapenems		Non-Carbapenem β-Lactams		Trimethoprim/ Sulfamethoxazole		Aminoglycosides	
**Resistance Genes**		**Received (n = 88)**	**Not Received (n = 78)**	**Received (n = 73)**	**Not Received (n = 93)**	**Received (n = 115)**	**Not Received (n = 51)**	**Received (n = 107)**	**Not Received (n = 59)**
***bla*$_{CTX-M-1-Family}$**	**Positive**	50 (56.8%)	33 (42.3%)	23 (31.5%)	60 (64.5%)	70 (60.9%)	13 (25.5%)	46 (43%)	37 (62.7%)
	**Negative**	38 (43.2%)	45 (57.7%)	50 (68.5%)	33 (35.5%)	45 (39.1%)	38 (74.5%)	61 (57%)	22 (37.3%)
***bla*$_{OXA-1}$**	**Positive**	43 (48.9%)	28 (36.4%)	21 (28.8%)	50 (53.8%)	60 (52.2%)	11 (21.6%)	39 (36.4%)	32 (54.2%)
	**Negative**	45 (51.1%)	50 (63.6%)	52 (71.2%)	43 (46.2%)	55 (47.8%)	40 (78.4%)	68 (63.6%)	27 (45.8%)
***bla*$_{OXA-48}$**	**Positive**	46 (52.3%)	28 (35.9%)	23 (31.5%)	51 (54.8%)	67 (58.3%)	7 (13.7%)	46 (43%)	28 (47.5%)
	**Negative**	42 (47.7%)	50 (64.1%)	50 (68.5%)	42 (45.2%)	48 (41.7%)	44 (86.3%)	61 (57%)	31 (52.5%)
***bla*$_{VIM}$**	**Positive**	36 (40.9%)	27 (34.6%)	19 (26.1%)	44 (47.3%)	51 (44.3%)	12 (23.5%)	37 (34.6%)	26 (44.1%)
	**Negative**	52 (59.1%)	51 (65.4%)	54 (73.9%)	49 (52.7%)	64 (55.7%)	39 (76.5%)	70 (65.4%)	33 (55.9%)

Supplementary Table 2. Relative intestinal loads of *bla*$_{CTX-M-1-Family}$, *bla*$_{OXA-1}$, *bla*$_{OXA-48}$, and *bla*$_{VIM}$ of the positive samples in relation to whether or not the patients received carbapenems, non-carbapenem β-lactams, trimethoprim/sulfamethoxazole, and/or aminoglycosides within the past 30 days. Three samples were not included in this analysis since the antibiotic consumption data was not available in the information system database. "%RL" stands for the percent relative load.

		**Carbapenems**		**Non-Carbapenem β-Lactams**		**Trimethoprim/ Sulfamethoxazole**		**Aminoglycosides**	
**Gene**	**%RL**	**Received (n = 50)**	**Not Received (n = 33)**	**Received (n = 23)**	**Not Received (n = 60)**	**Received (n = 70)**	**Not Received (n = 13)**	**Received (n = 46)**	**Not Received (n = 37)**
***bla*$_{CTX-M-1-Family}$**	**Very High**	15 (30%)	5 (15.2%)	8 (34.8%)	12 (20%)	18 (25.7%)	2 (15.4%)	17 (37%)	3 (8.1%)
	**High**	29 (58%)	18 (54.5%)	11 (47.8%)	36 (60%)	41 (58.6%)	6 (46.2%)	21 (45.7%)	26 (70.3%)
	**Low**	6 (12%)	10 (30.3%)	4 (17.4%)	12 (20%)	11 (15.7%)	5 (38.4%)	8 (17.3%)	8 (21.6%)
**Gene**	**%RL**	**Received (n = 43)**	**Not Received (n = 28)**	**Received (n = 21)**	**Not Received (n = 50)**	**Received (n = 60)**	**Not Received (n = 11)**	**Received (n = 39)**	**Not Received (n = 32)**
***bla*$_{OXA-1}$**	**Very High**	15 (34.9%)	3 (10.7%)	8 (38.1%)	10 (20%)	16 (26.7%)	2 (18.2%)	15 (38.5%)	3 (9.4%)
	**High**	23 (53.5%)	15 (53.6%)	12 (57.1%)	26 (52%)	32 (53.3%)	6 (54.5%)	17 (43.6%)	21 (65.6%)
	**Low**	5 (11.6%)	10 (35.7%)	1 (4.8%)	14 (28%)	12 (20%)	3 (27.3%)	7 (17.9%)	8 (25%)
**Gene**	**%RL**	**Received (n = 46)**	**Not Received (n = 28)**	**Received (n = 23)**	**Not Received (n = 51)**	**Received (n = 67)**	**Not Received (n = 7)**	**Received (n = 46)**	**Not Received (n = 28)**
***bla*$_{OXA-48}$**	**Very High**	13 (28.3%)	8 (28.6%)	6 (26.1%)	15 (29.4%)	19 (28.4%)	2 (28.6%)	15 (32.6%)	6 (21.4%)
	**High**	27 (58.7%)	9 (32.1%)	11 (47.8%)	25 (49%)	36 (53.7%)	0 (0%)	21 (45.7%)	15 (53.6%)
	**Low**	6 (13%)	11 (39.3%)	6 (26.1%)	11 (21.6%)	12 (17.9%)	5 (71.4%)	10 (21.7%)	7 (25%)
**Gene**	**%RL**	**Received (n = 36)**	**Not Received (n = 27)**	**Received (n = 19)**	**Not Received (n = 44)**	**Received (n = 51)**	**Not Received (n = 12)**	**Received (n = 37)**	**Not Received (n = 26)**
***bla*$_{VIM}$**	**Very High**	8 (22.2%)	5 (18.5%)	5 (26.3%)	8 (18.2%)	11 (21.6%)	2 (16.7%)	10 (27%)	3 (11.5%)
	**High**	18 (50%)	8 (29.6%)	5 (26.3%)	21 (47.7%)	23 (45.1%)	3 (25%)	15 (40.6%)	11 (42.3%)
	**Low**	10 (27.8%)	14 (51.9%)	9 (47.4%)	15 (34.1%)	17 (33.3%)	7 (58.3%)	12 (32.4%)	12 (46.2%)

Supplementary File 1. Graphs of the relative intestinal loads of the antibiotic resistance genes for all the patients included in this study.

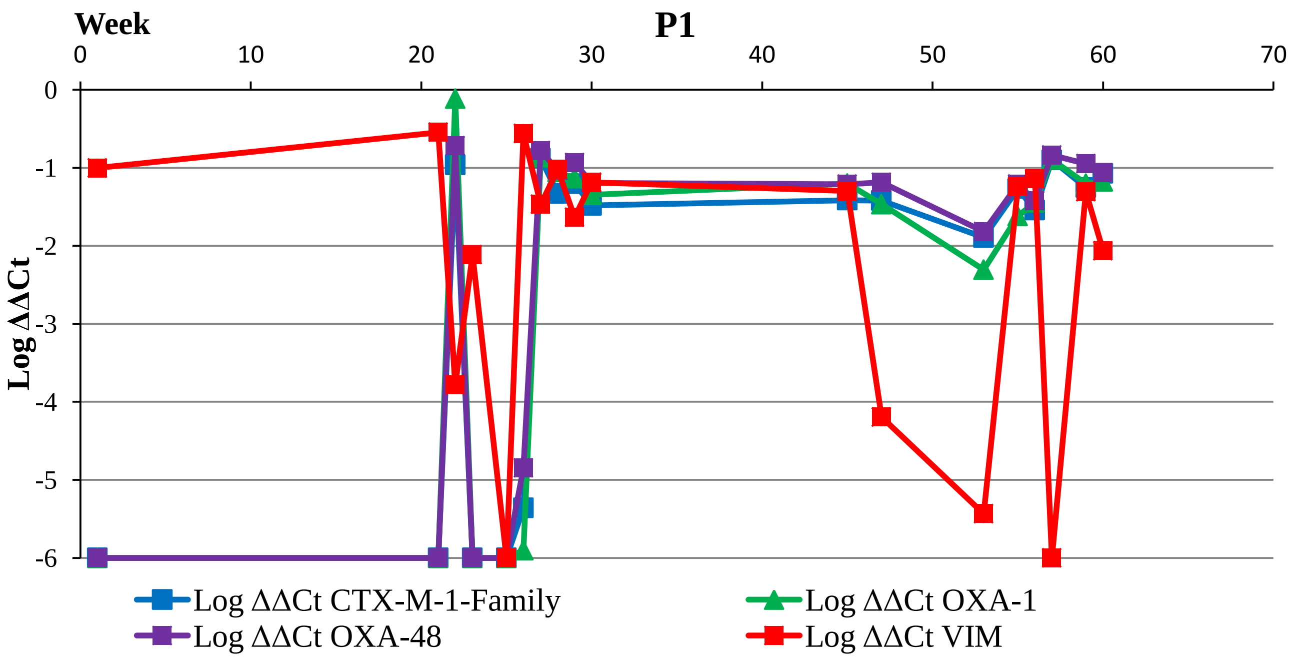

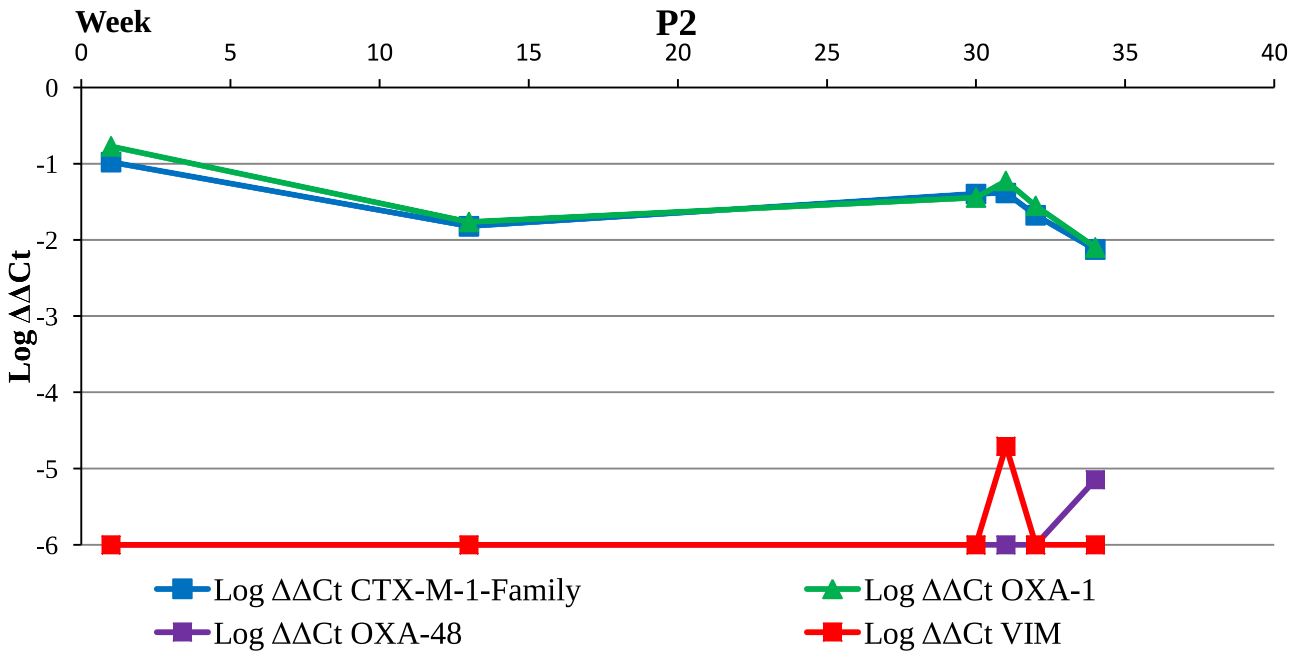

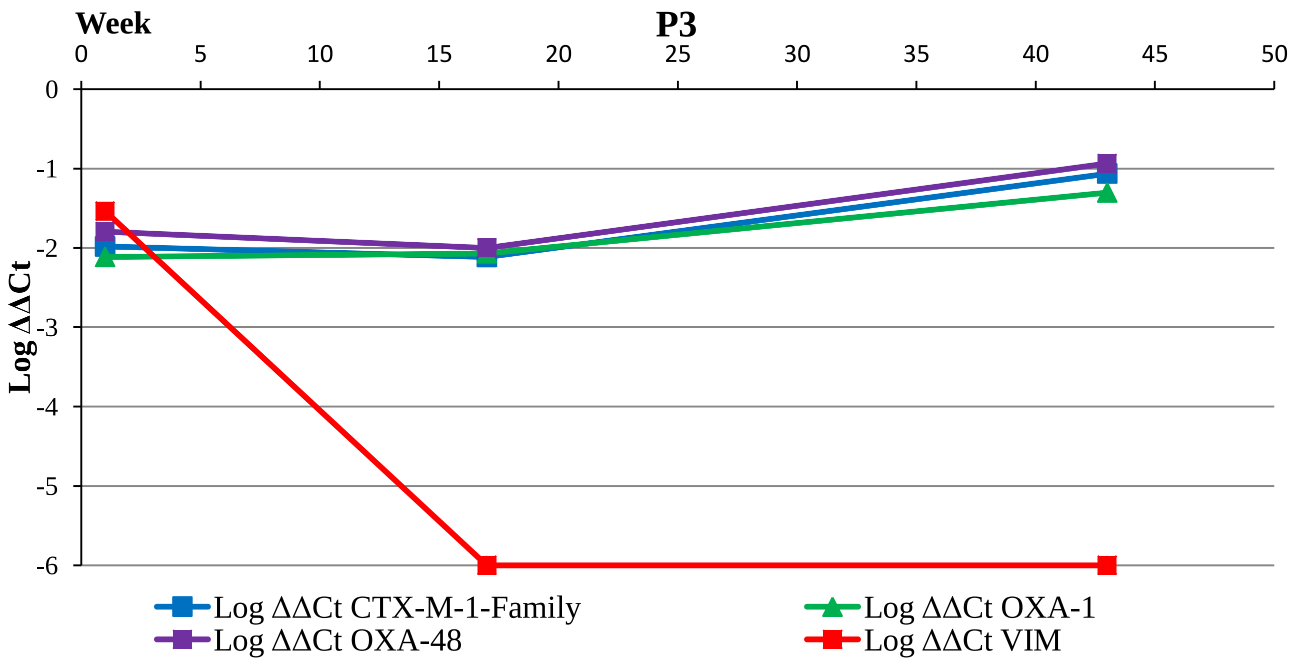

Week
P3
0
5
10
15
20
25
30
35
40
45
50
0
-1
-2
-3
-4
-5
-6
Log ΔΔCt
Log ΔΔCt CTX-M-1-Family
Log ΔΔCt OXA-1
Log ΔΔCt OXA-48
Log ΔΔCt VIM

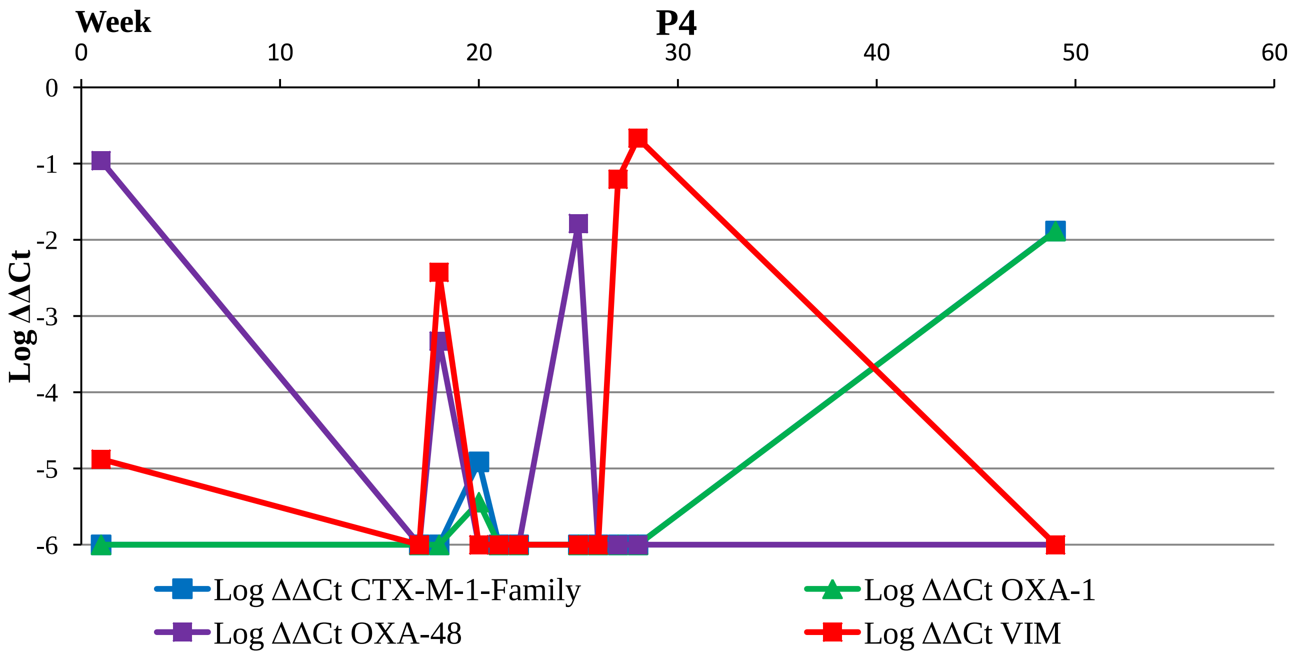

Week
P4
0
10
20
30
40
50
60
0
-1
-2
-3
-4
-5
-6
Log ΔΔCt
Log ΔΔCt CTX-M-1-Family
Log ΔΔCt OXA-1
Log ΔΔCt OXA-48
Log ΔΔCt VIM

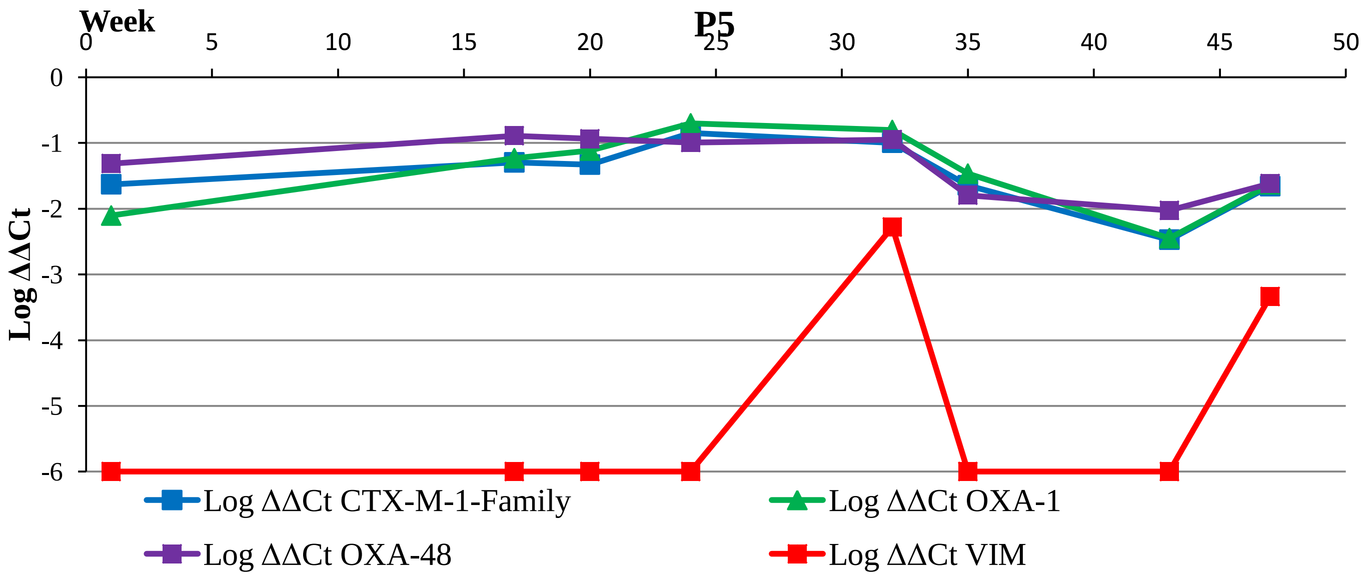

P5
Week
0
5
10
15
20
25
30
35
40
45
50
Log ΔΔCt
0
-1
-2
-3
-4
-5
-6
Log ΔΔCt CTX-M-1-Family
Log ΔΔCt OXA-1
Log ΔΔCt OXA-48
Log ΔΔCt VIM

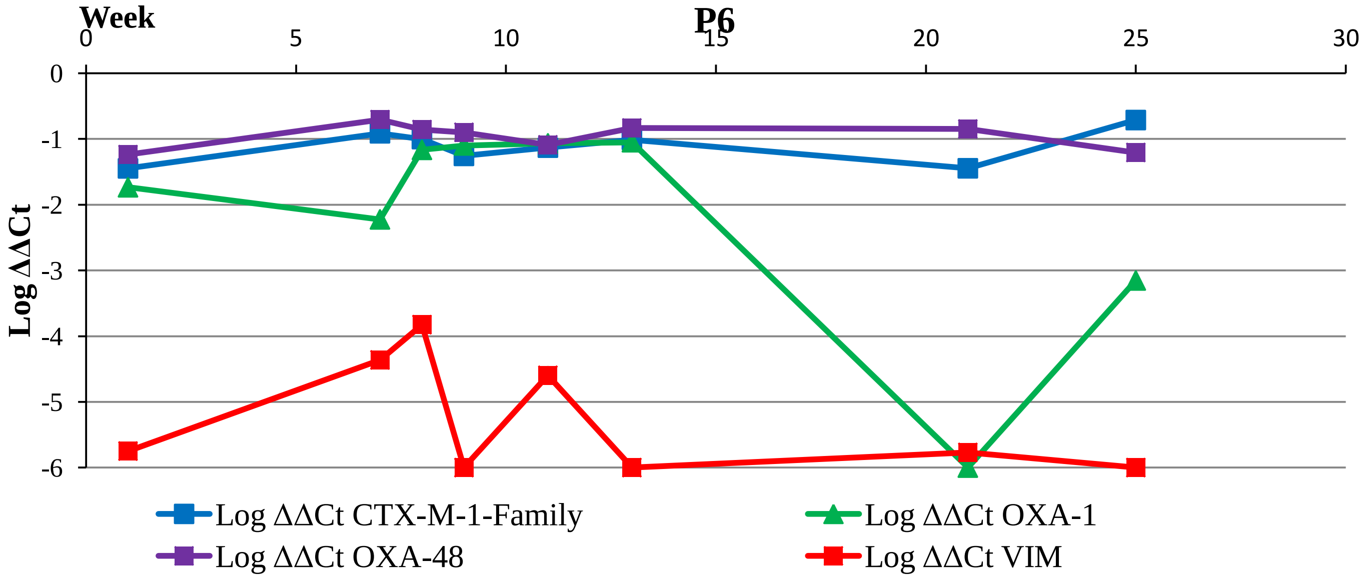

P6
Week
0
5
10
15
20
25
30
Log ΔΔCt
0
-1
-2
-3
-4
-5
-6
Log ΔΔCt CTX-M-1-Family
Log ΔΔCt OXA-1
Log ΔΔCt OXA-48
Log ΔΔCt VIM

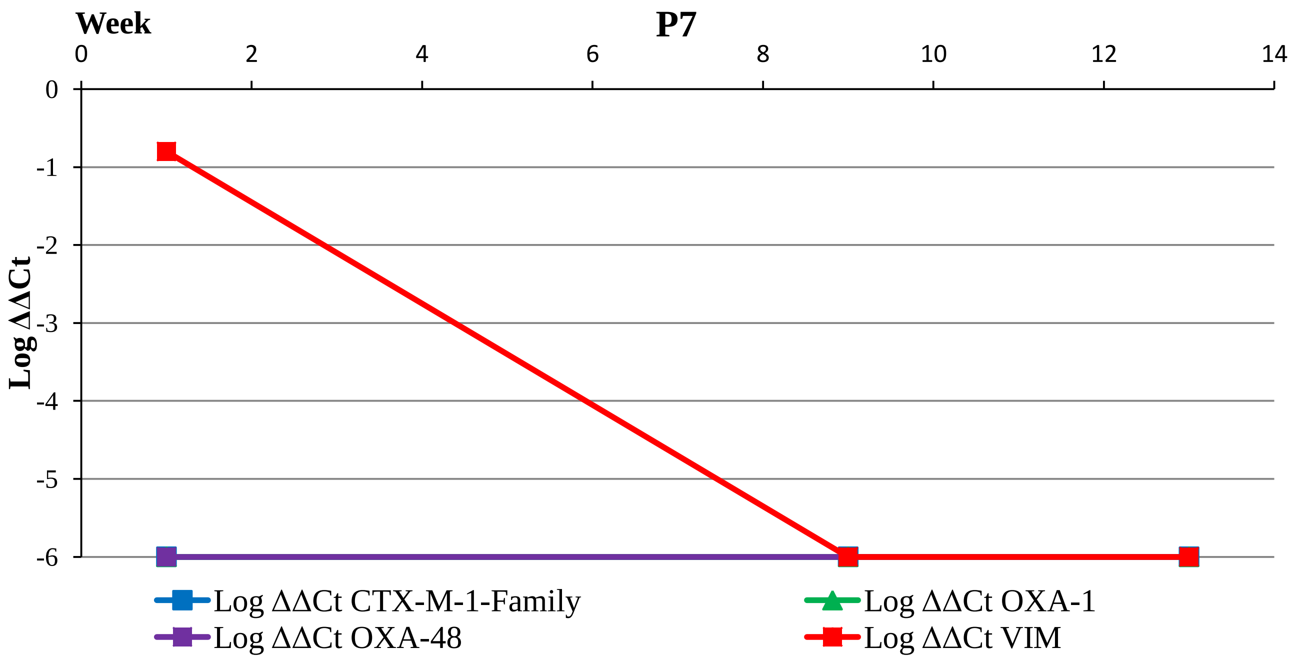
P7
Week
0
2
4
6
8
10
12
14
0
-1
-2
-3
-4
-5
-6
Log ΔΔCt
Log ΔΔCt CTX-M-1-Family
Log ΔΔCt OXA-1
Log ΔΔCt OXA-48
Log ΔΔCt VIM

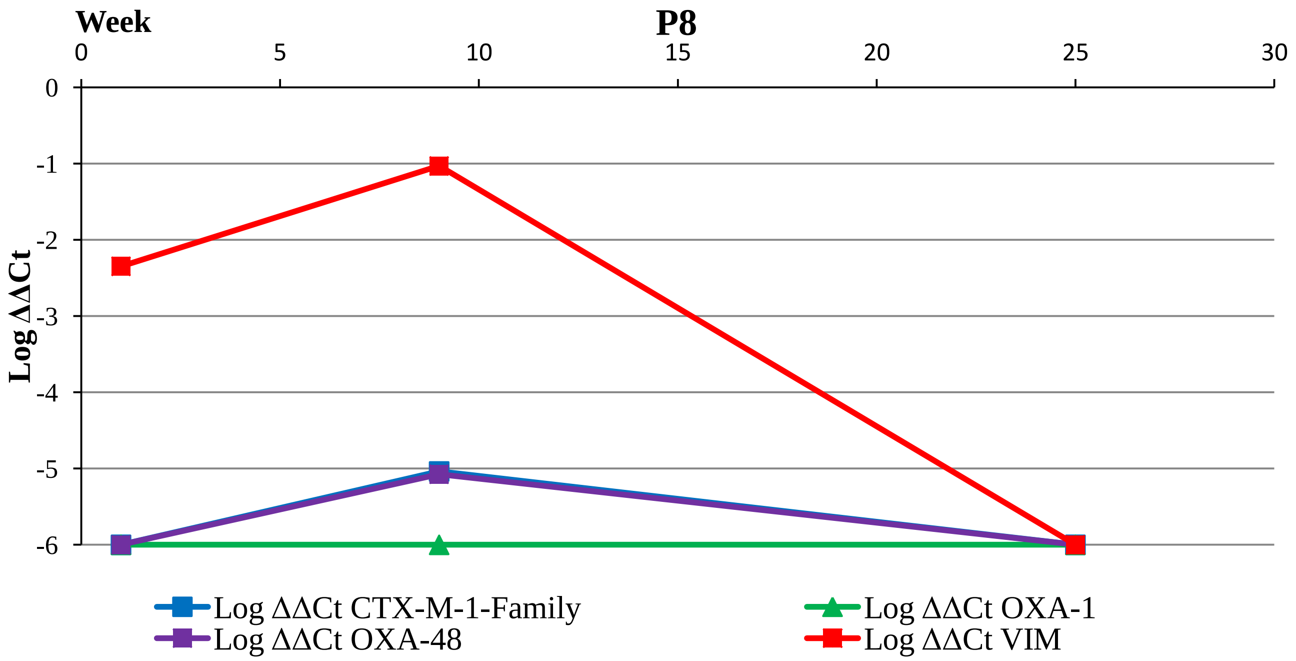
P8
Week
0
5
10
15
20
25
30
0
-1
-2
-3
-4
-5
-6
Log ΔΔCt
Log ΔΔCt CTX-M-1-Family
Log ΔΔCt OXA-1
Log ΔΔCt OXA-48
Log ΔΔCt VIM

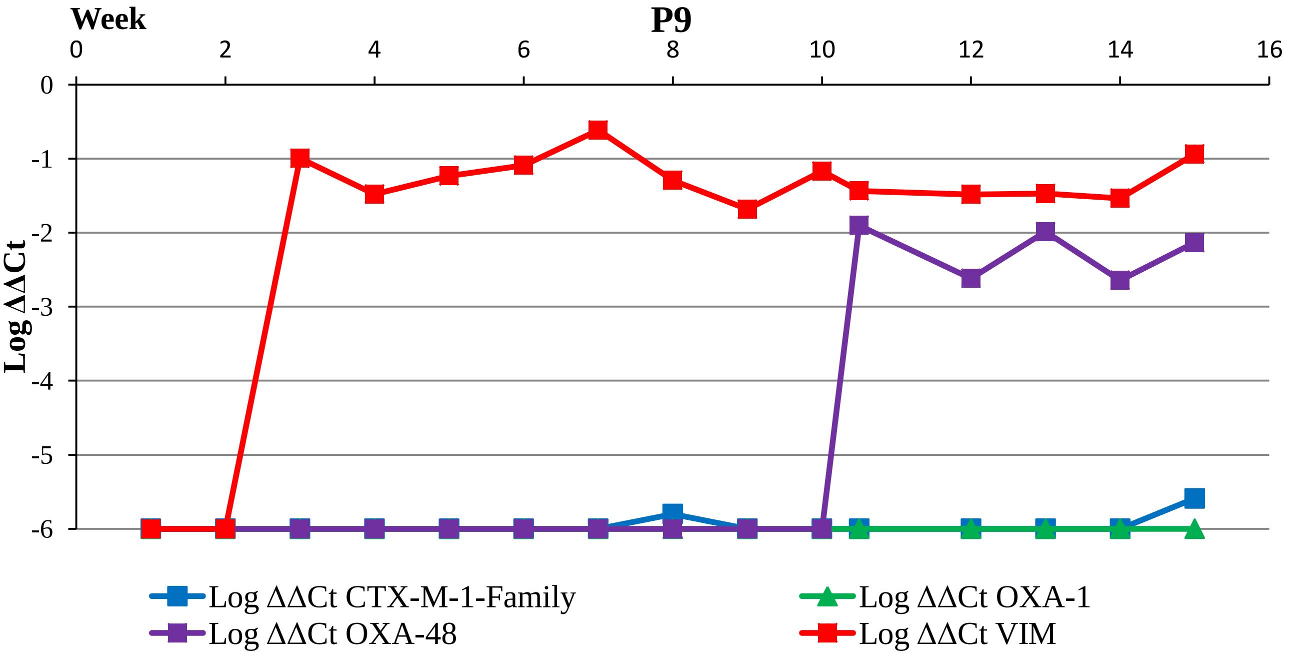
Week
P9
0
2
4
6
8
10
12
14
16
0
-1
-2
-3
-4
-5
-6
Log ΔΔCt
Log ΔΔCt CTX-M-1-Family
Log ΔΔCt OXA-1
Log ΔΔCt OXA-48
Log ΔΔCt VIM

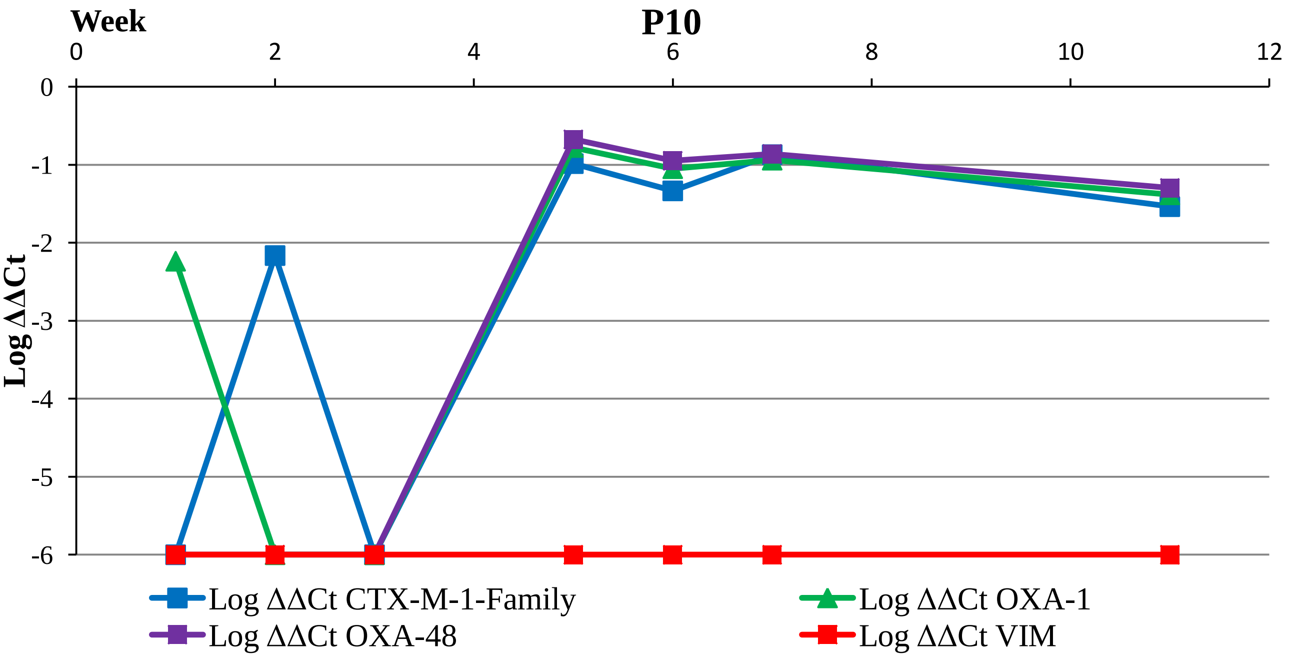
Week
P10
0
2
4
6
8
10
12
0
-1
-2
-3
-4
-5
-6
Log ΔΔCt
Log ΔΔCt CTX-M-1-Family
Log ΔΔCt OXA-1
Log ΔΔCt OXA-48
Log ΔΔCt VIM

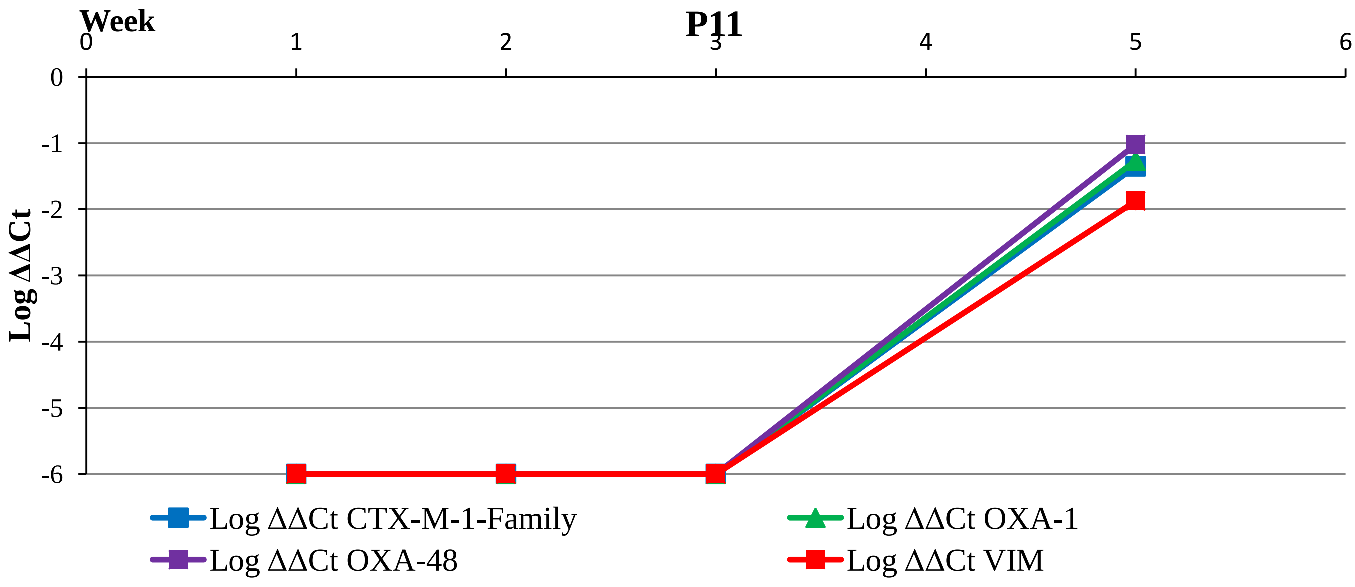
P11
Week
0
1
2
3
4
5
6
0
-1
-2
-3
-4
-5
-6
Log ΔΔCt
Log ΔΔCt CTX-M-1-Family
Log ΔΔCt OXA-1
Log ΔΔCt OXA-48
Log ΔΔCt VIM

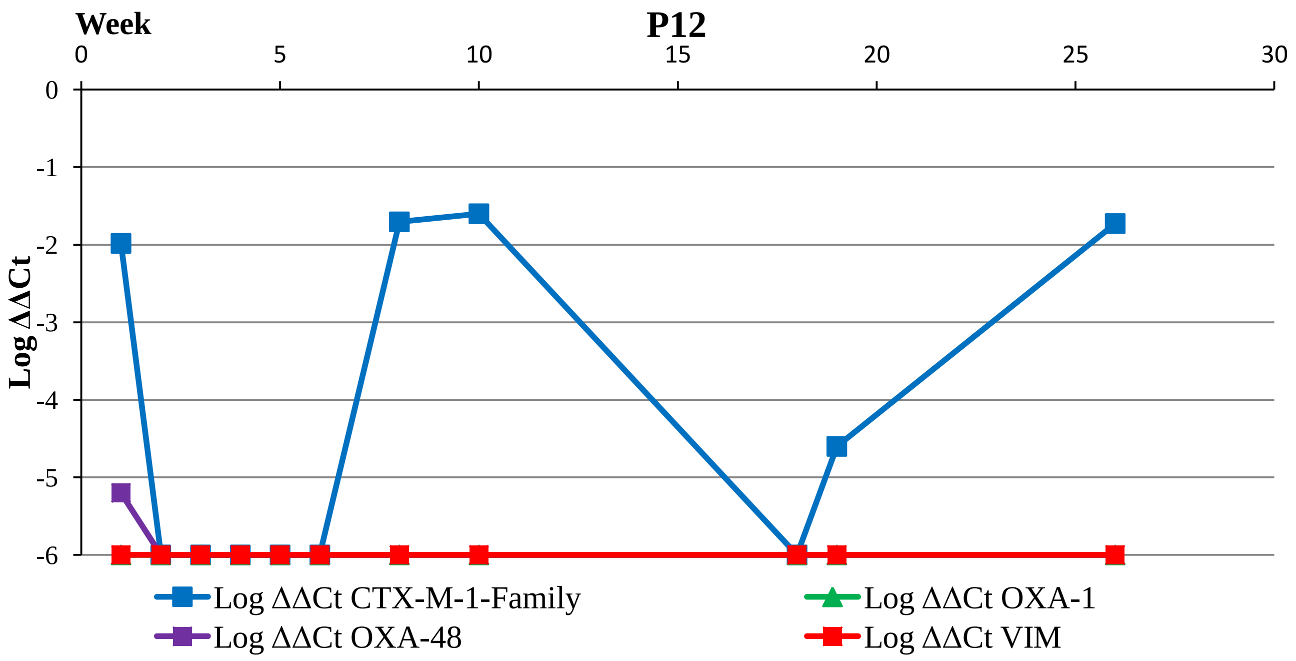
P12
Week
0
5
10
15
20
25
30
0
-1
-2
-3
-4
-5
-6
Log ΔΔCt
Log ΔΔCt CTX-M-1-Family
Log ΔΔCt OXA-1
Log ΔΔCt OXA-48
Log ΔΔCt VIM

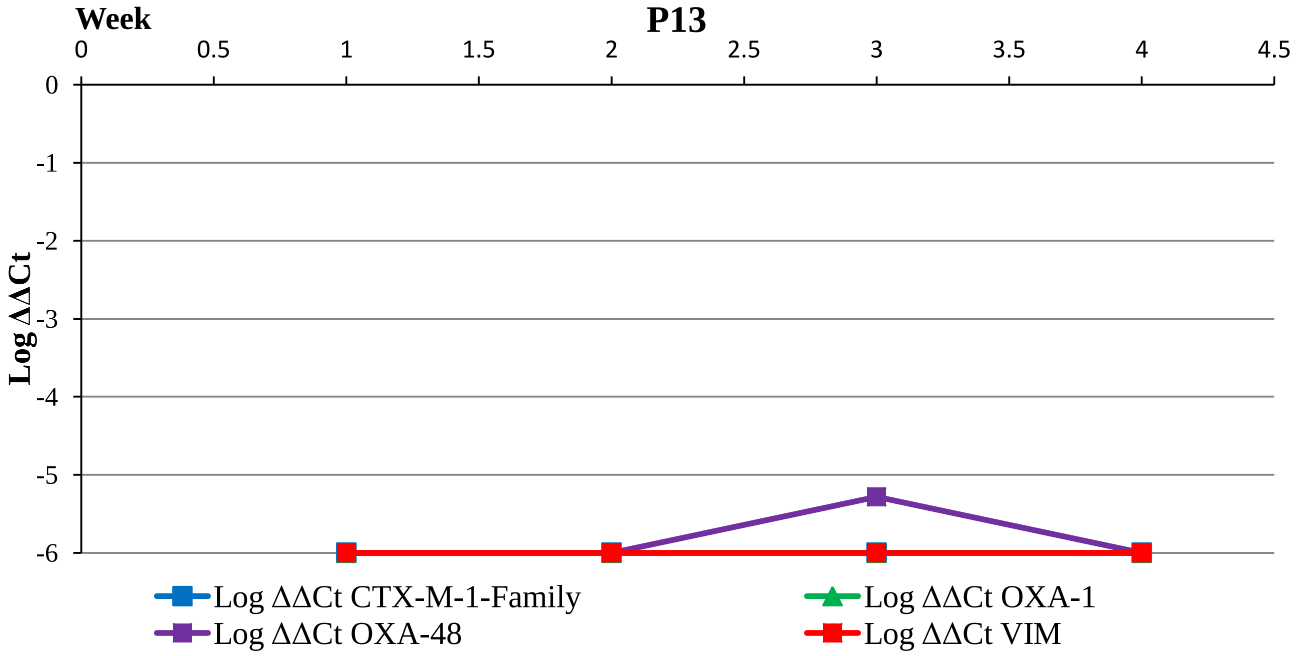

P13
Week
0
0.5
1
1.5
2
2.5
3
3.5
4
4.5
Log ΔΔCt
0
-1
-2
-3
-4
-5
-6
Log ΔΔCt CTX-M-1-Family
Log ΔΔCt OXA-1
Log ΔΔCt OXA-48
Log ΔΔCt VIM

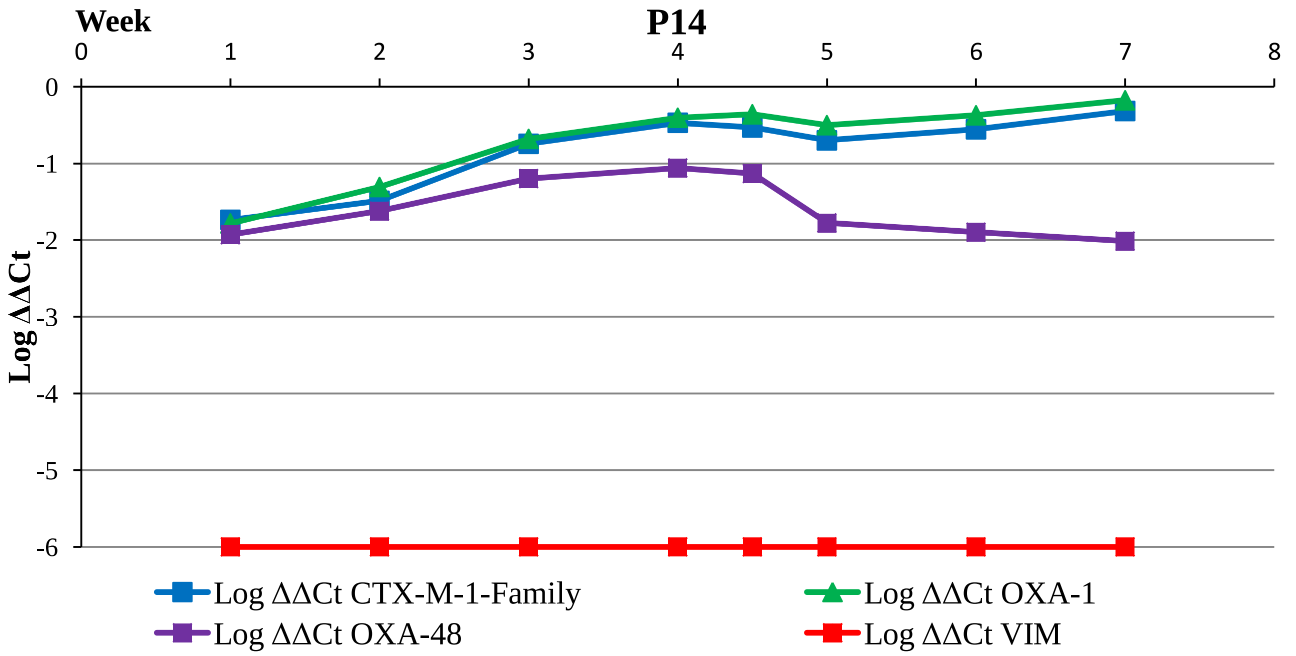

P14
Week
0
1
2
3
4
5
6
7
8
Log ΔΔCt
0
-1
-2
-3
-4
-5
-6
Log ΔΔCt CTX-M-1-Family
Log ΔΔCt OXA-1
Log ΔΔCt OXA-48
Log ΔΔCt VIM

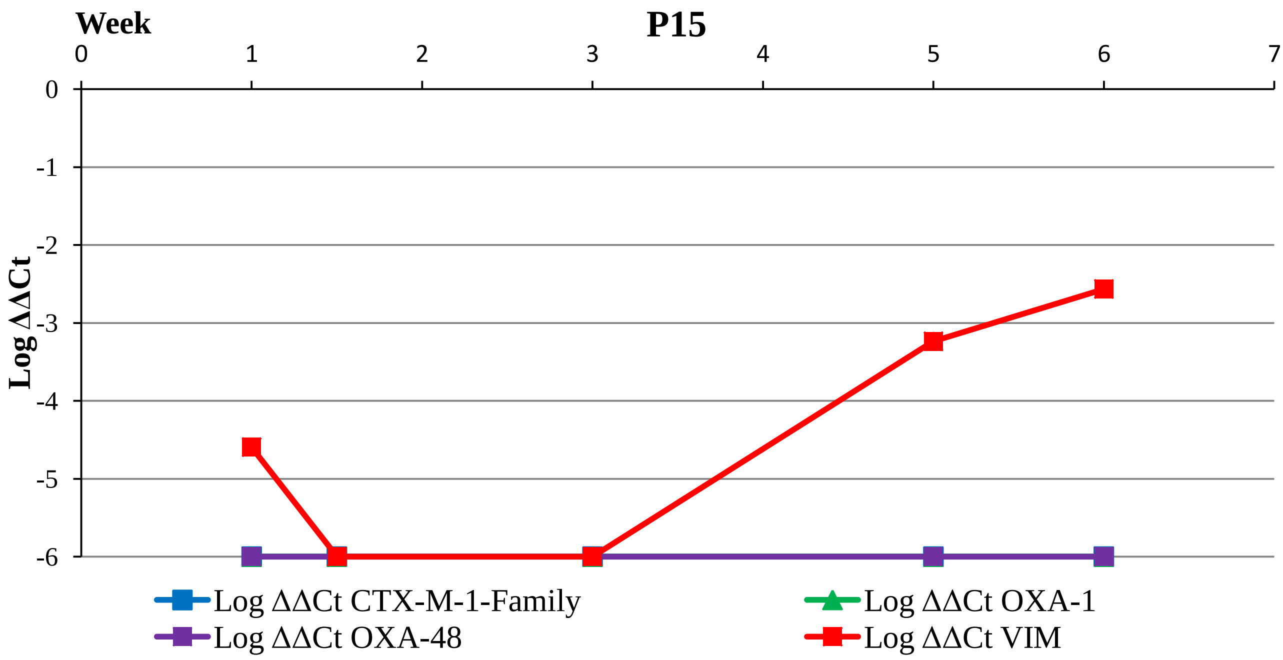
Week
P15
0
1
2
3
4
5
6
7
0
-1
-2
-3
-4
-5
-6
Log ΔΔCt
Log ΔΔCt CTX-M-1-Family
Log ΔΔCt OXA-1
Log ΔΔCt OXA-48
Log ΔΔCt VIM

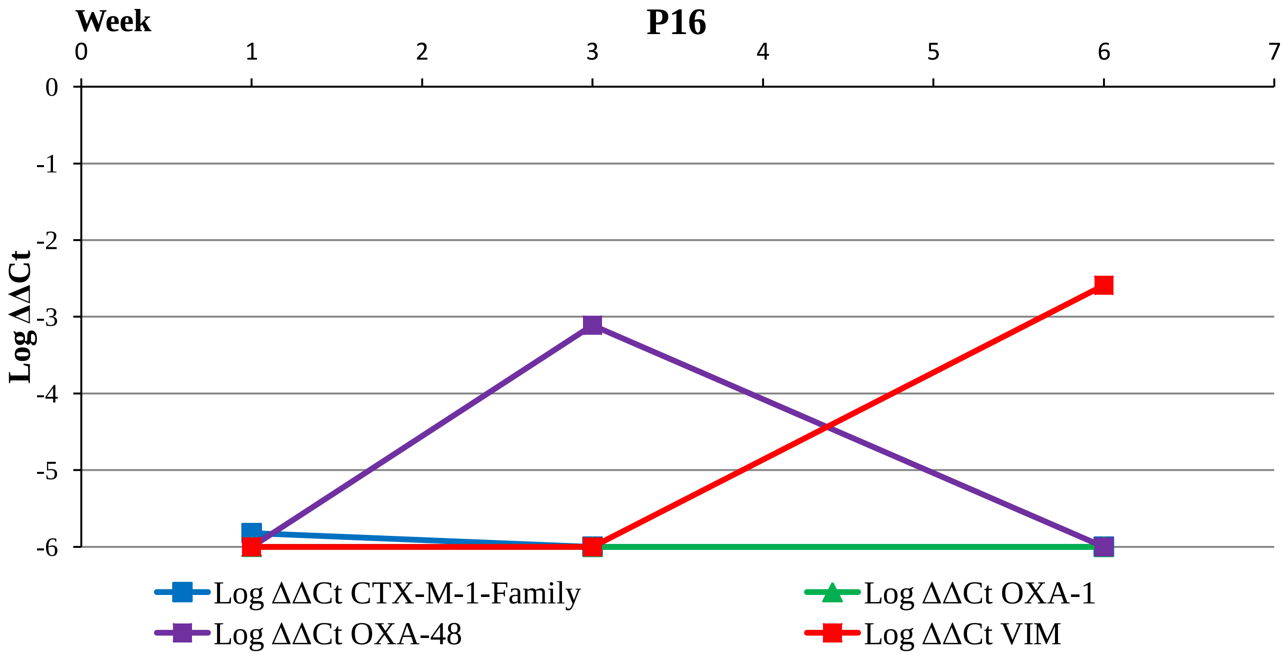
Week
P16
0
1
2
3
4
5
6
7
0
-1
-2
-3
-4
-5
-6
Log ΔΔCt
Log ΔΔCt CTX-M-1-Family
Log ΔΔCt OXA-1
Log ΔΔCt OXA-48
Log ΔΔCt VIM

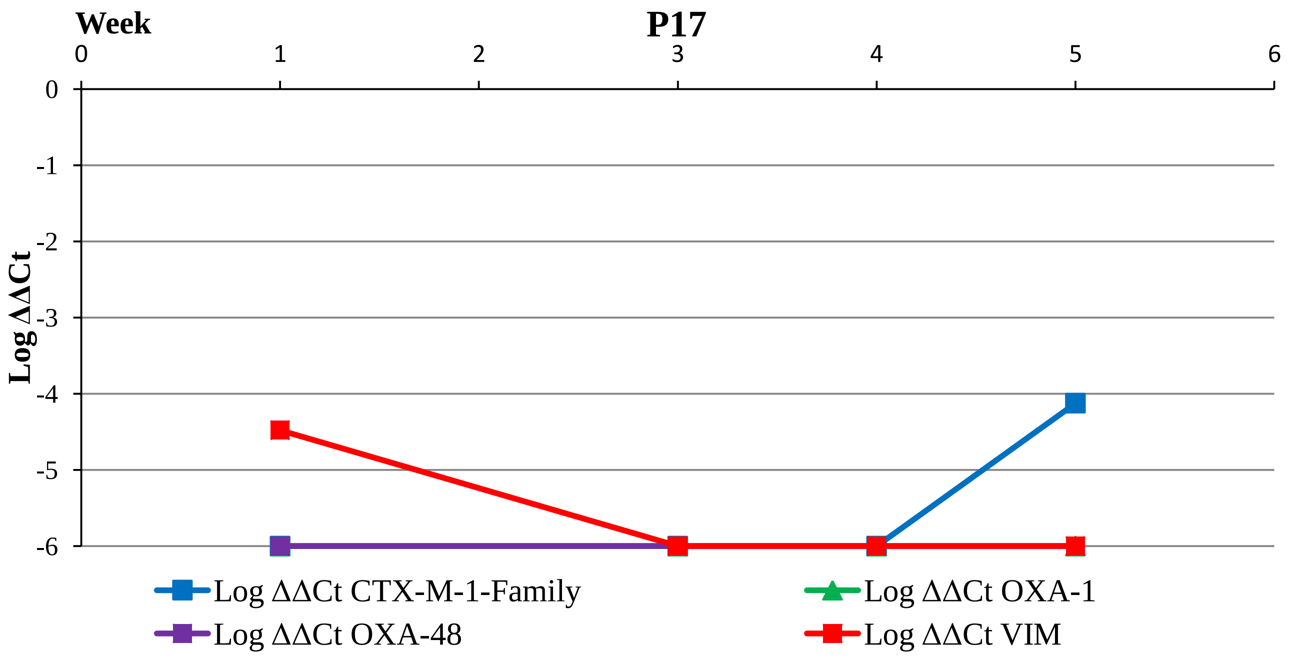

Week
P17
0 1 2 3 4 5 6
0 -1 -2 -3 -4 -5 -6
Log ΔΔCt
Log ΔΔCt CTX-M-1-Family
Log ΔΔCt OXA-1
Log ΔΔCt OXA-48
Log ΔΔCt VIM

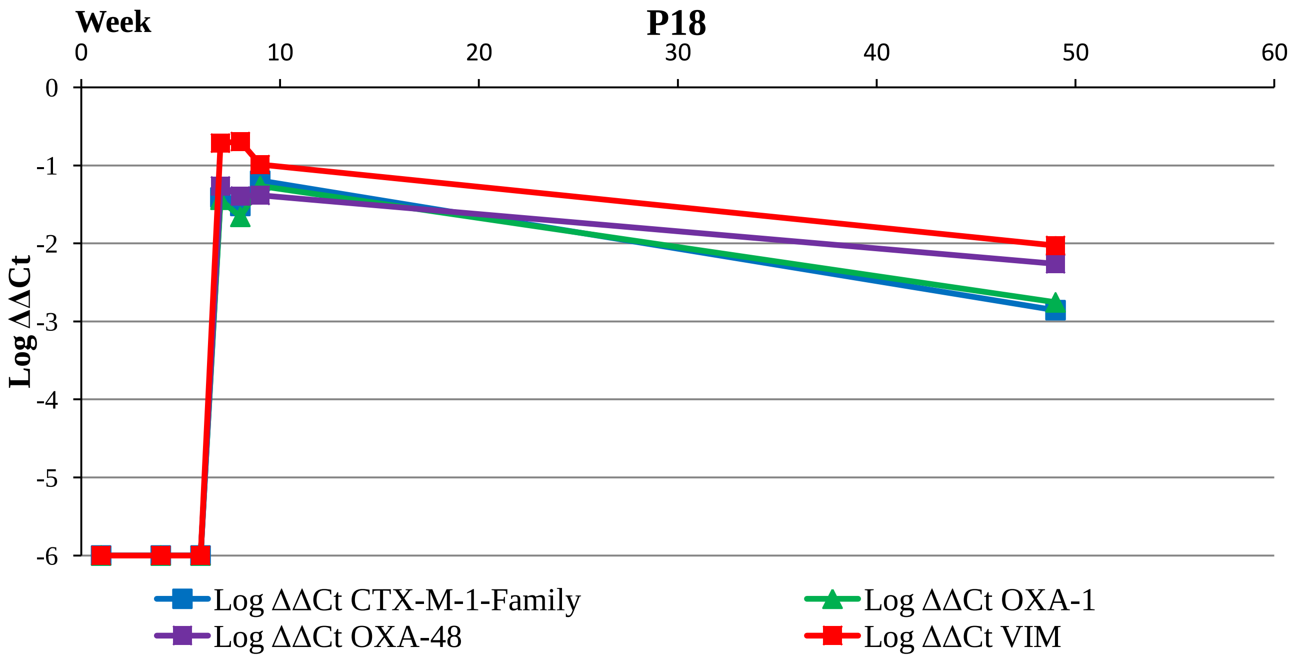

Week
P18
0 10 20 30 40 50 60
0 -1 -2 -3 -4 -5 -6
Log ΔΔCt
Log ΔΔCt CTX-M-1-Family
Log ΔΔCt OXA-1
Log ΔΔCt OXA-48
Log ΔΔCt VIM

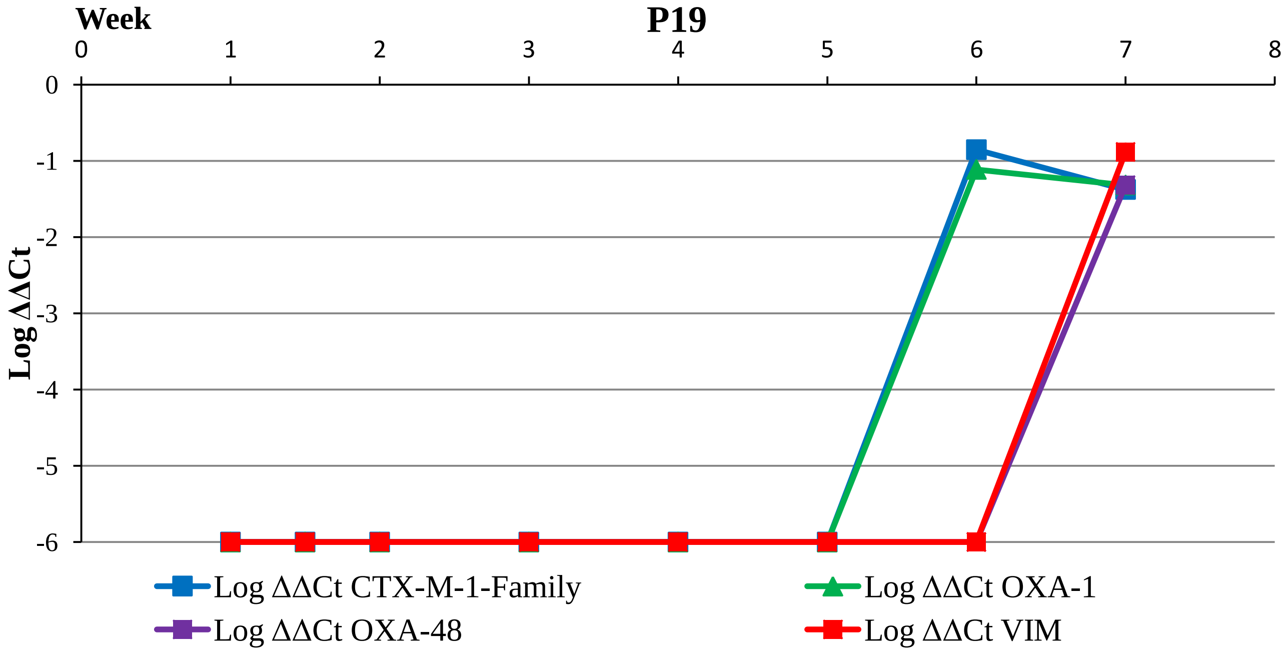

Week
P19
0
1
2
3
4
5
6
7
8
0
-1
-2
-3
-4
-5
-6
Log ΔΔCt
Log ΔΔCt CTX-M-1-Family
Log ΔΔCt OXA-1
Log ΔΔCt OXA-48
Log ΔΔCt VIM

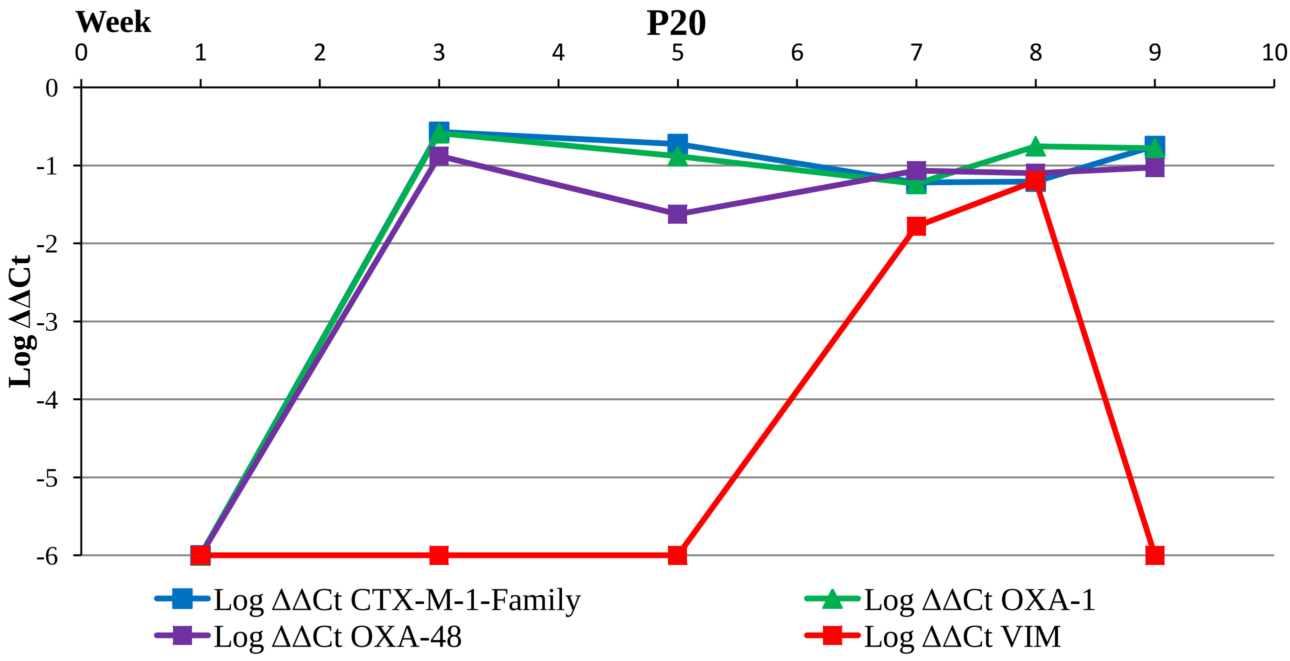

Week
P20
0
1
2
3
4
5
6
7
8
9
10
0
-1
-2
-3
-4
-5
-6
Log ΔΔCt
Log ΔΔCt CTX-M-1-Family
Log ΔΔCt OXA-1
Log ΔΔCt OXA-48
Log ΔΔCt VIM

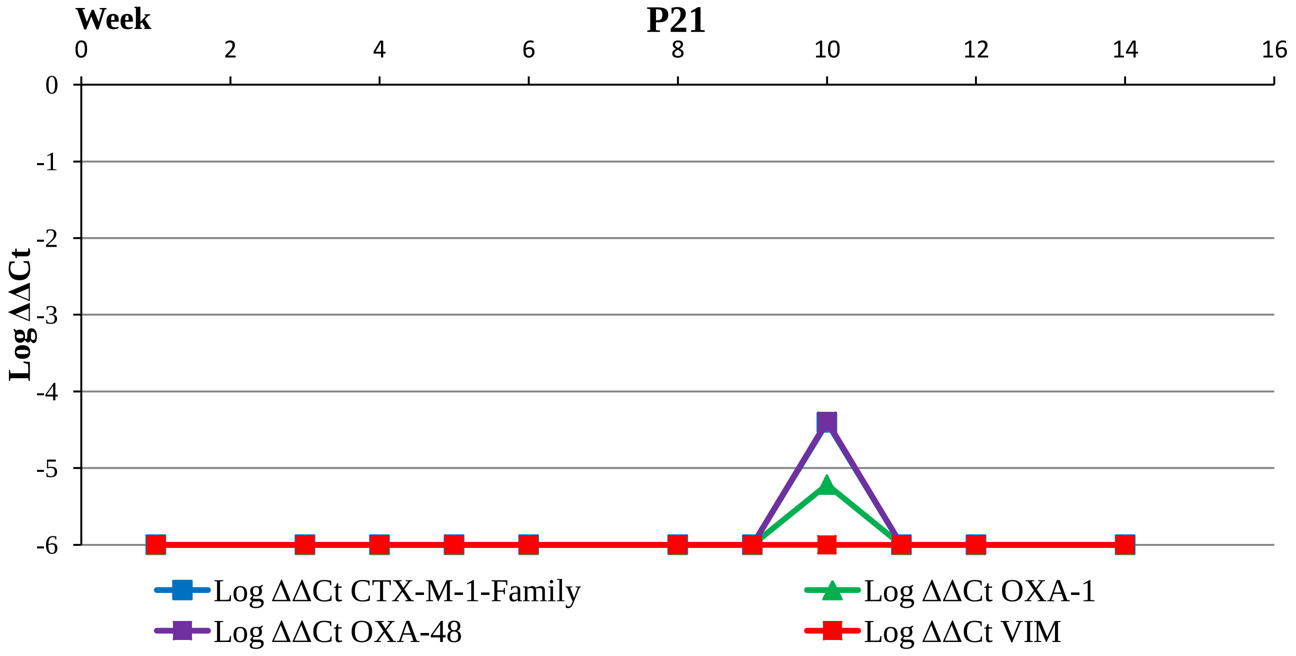

Week
P21
0 2 4 6 8 10 12 14 16
0 -1 -2 -3 -4 -5 -6
Log ΔΔCt
Log ΔΔCt CTX-M-1-Family
Log ΔΔCt OXA-1
Log ΔΔCt OXA-48
Log ΔΔCt VIM

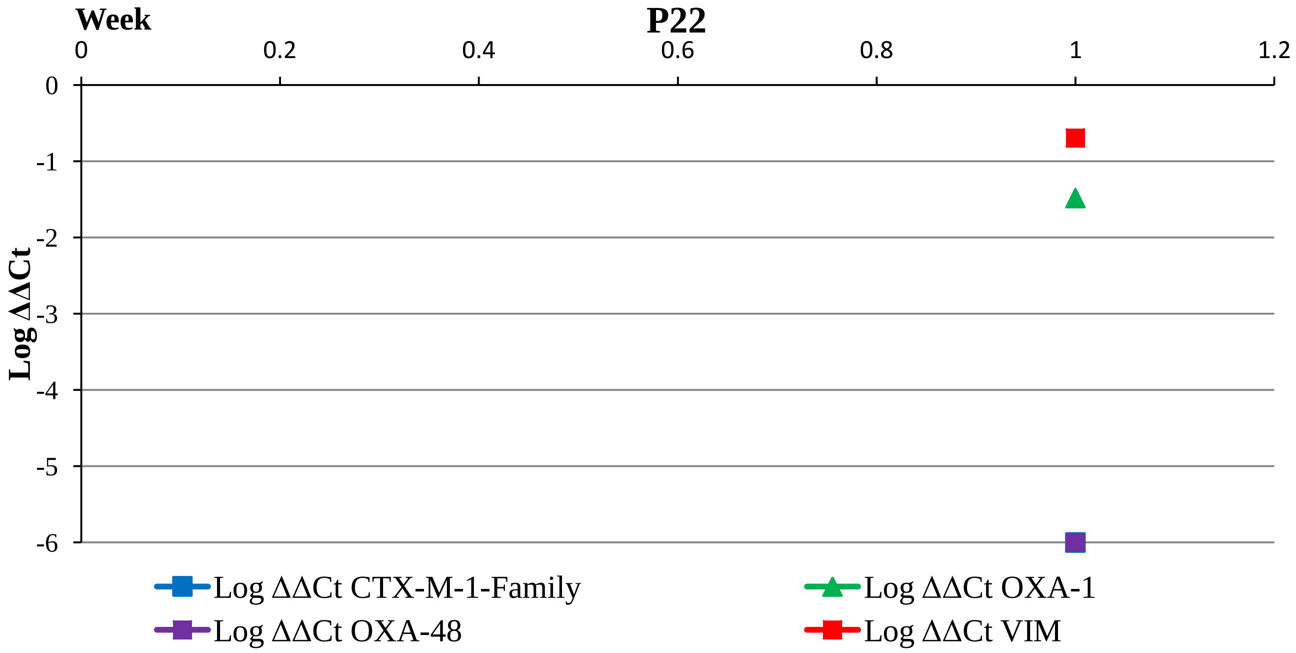

Week
P22
0 0.2 0.4 0.6 0.8 1 1.2
0 -1 -2 -3 -4 -5 -6
Log ΔΔCt
Log ΔΔCt CTX-M-1-Family
Log ΔΔCt OXA-1
Log ΔΔCt OXA-48
Log ΔΔCt VIM

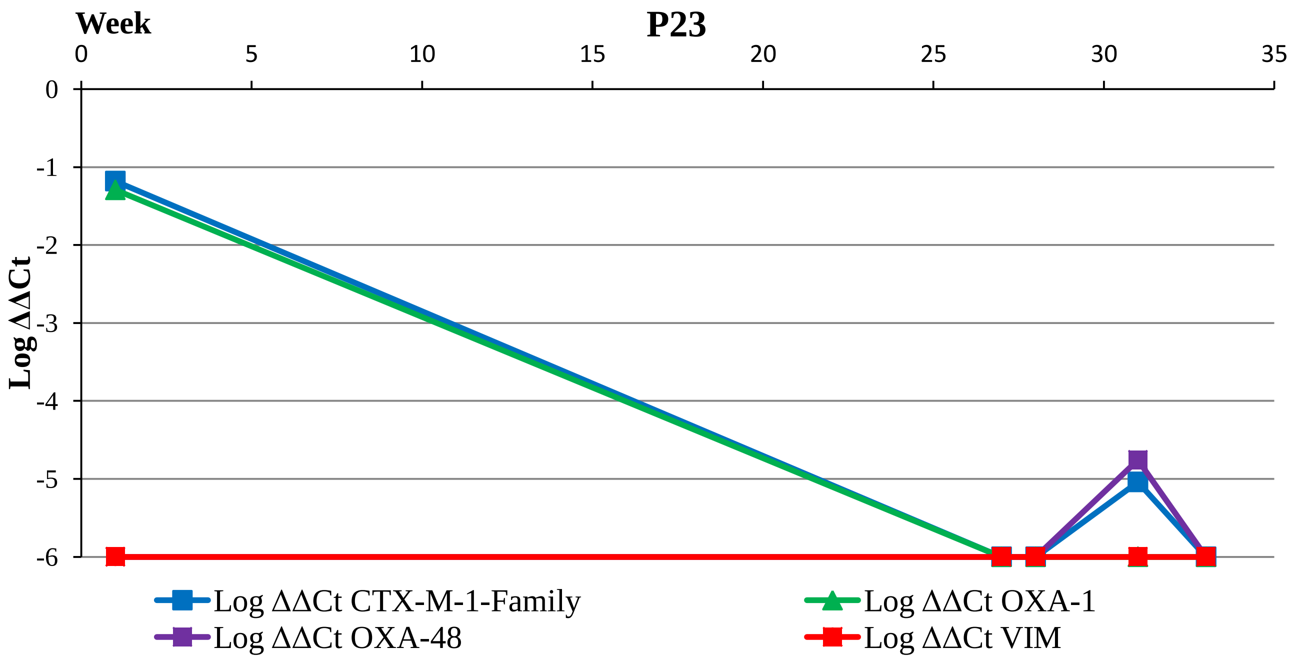
Week
P23
0
5
10
15
20
25
30
35
0
-1
-2
-3
-4
-5
-6
Log ΔΔCt
Log ΔΔCt CTX-M-1-Family
Log ΔΔCt OXA-1
Log ΔΔCt OXA-48
Log ΔΔCt VIM

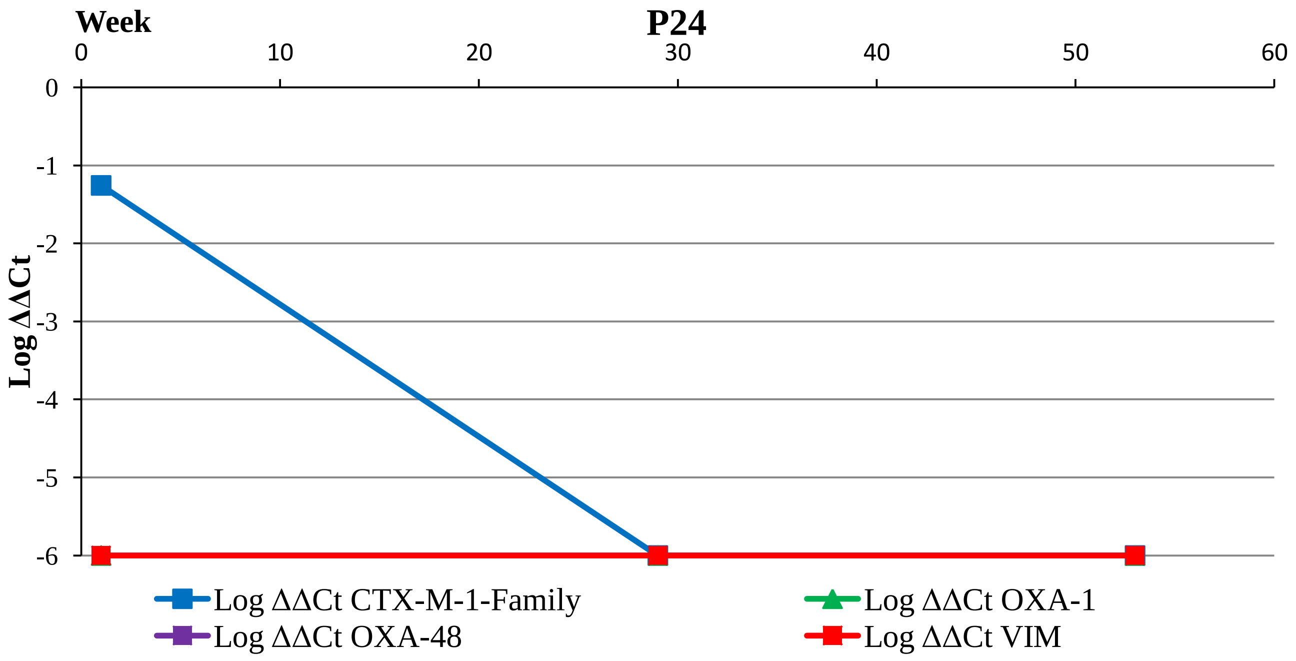
Week
P24
0
10
20
30
40
50
60
0
-1
-2
-3
-4
-5
-6
Log ΔΔCt
Log ΔΔCt CTX-M-1-Family
Log ΔΔCt OXA-1
Log ΔΔCt OXA-48
Log ΔΔCt VIM

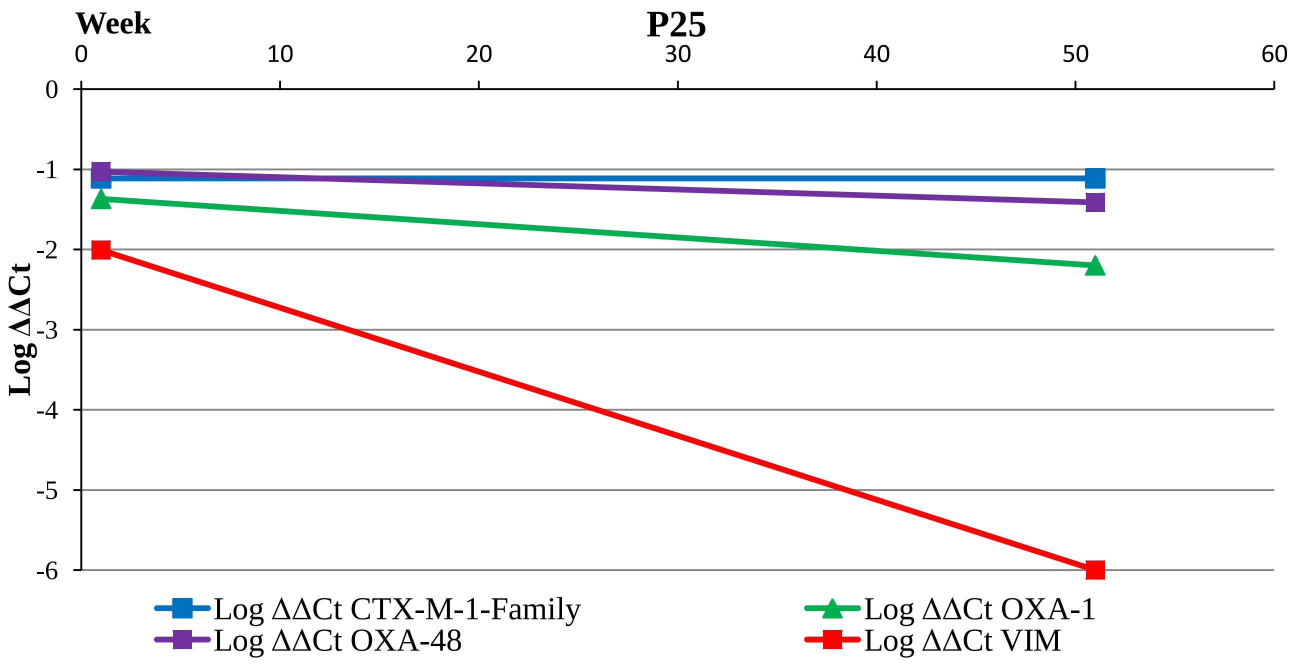

Week
P25
0
10
20
30
40
50
60
0
-1
-2
-3
-4
-5
-6
Log ΔΔCt
Log ΔΔCt CTX-M-1-Family
Log ΔΔCt OXA-1
Log ΔΔCt OXA-48
Log ΔΔCt VIM

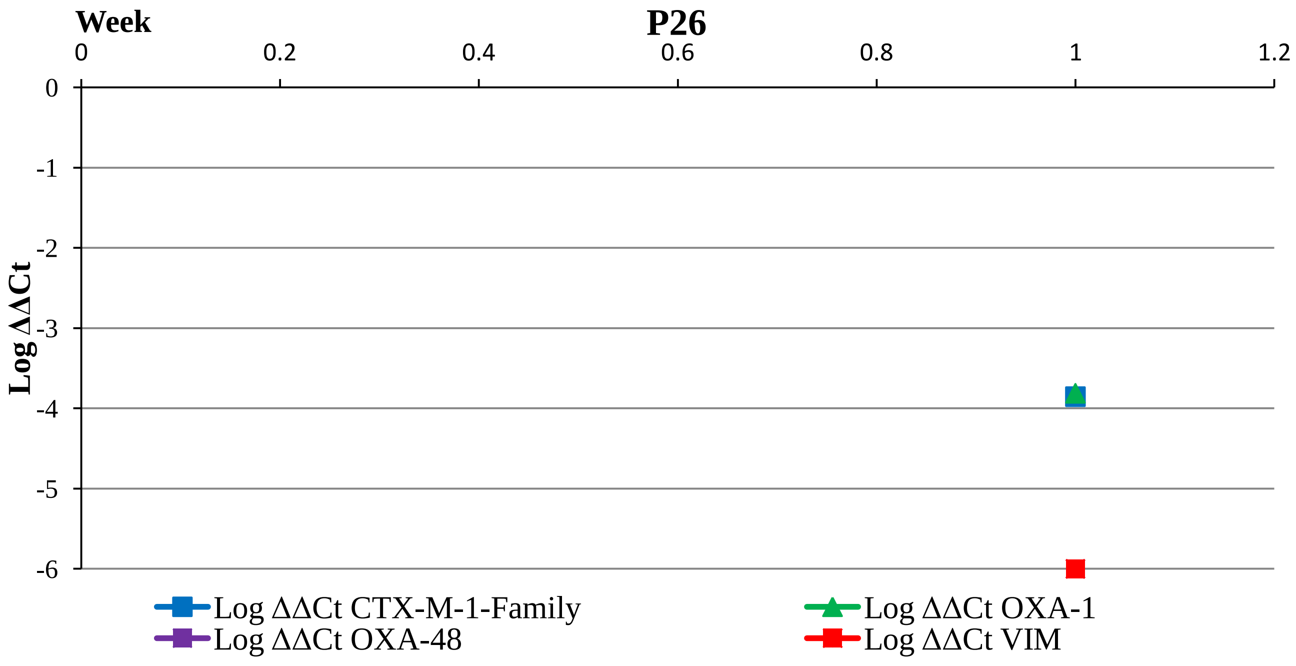

Week
P26
0
0.2
0.4
0.6
0.8
1
1.2
0
-1
-2
-3
-4
-5
-6
Log ΔΔCt
Log ΔΔCt CTX-M-1-Family
Log ΔΔCt OXA-1
Log ΔΔCt OXA-48
Log ΔΔCt VIM

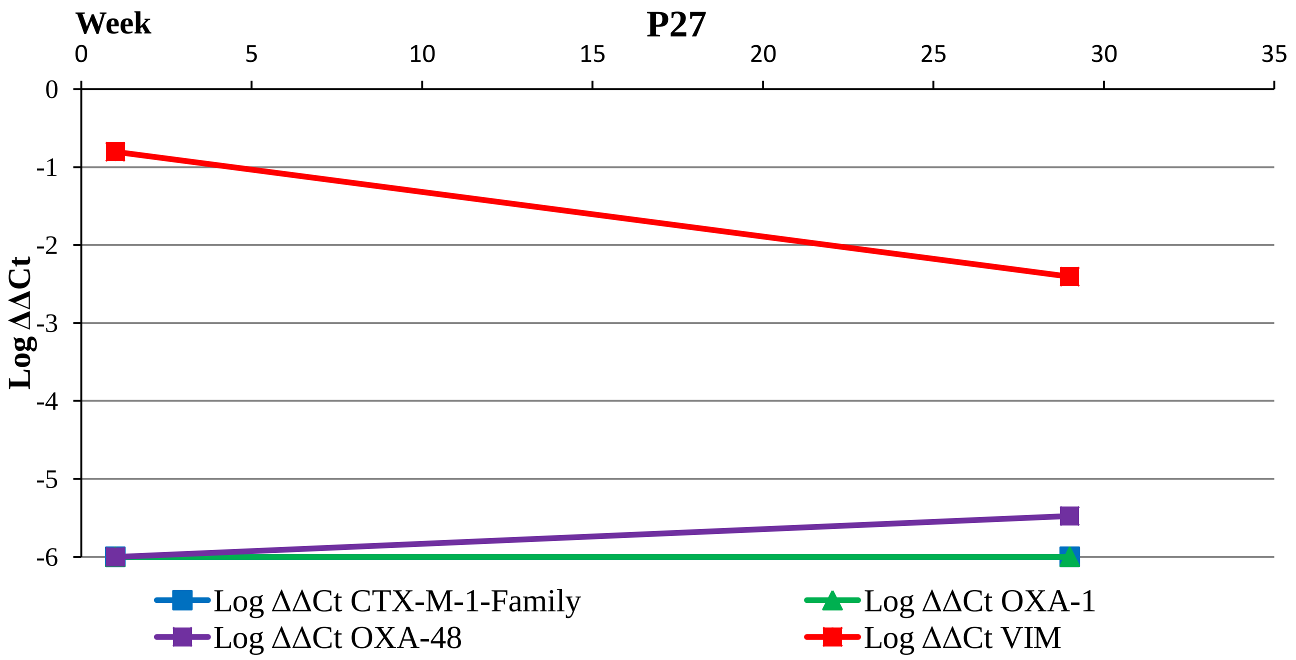
P27
Week
0
5
10
15
20
25
30
35
0
-1
-2
-3
-4
-5
-6
Log ΔΔCt
Log ΔΔCt CTX-M-1-Family
Log ΔΔCt OXA-1
Log ΔΔCt OXA-48
Log ΔΔCt VIM

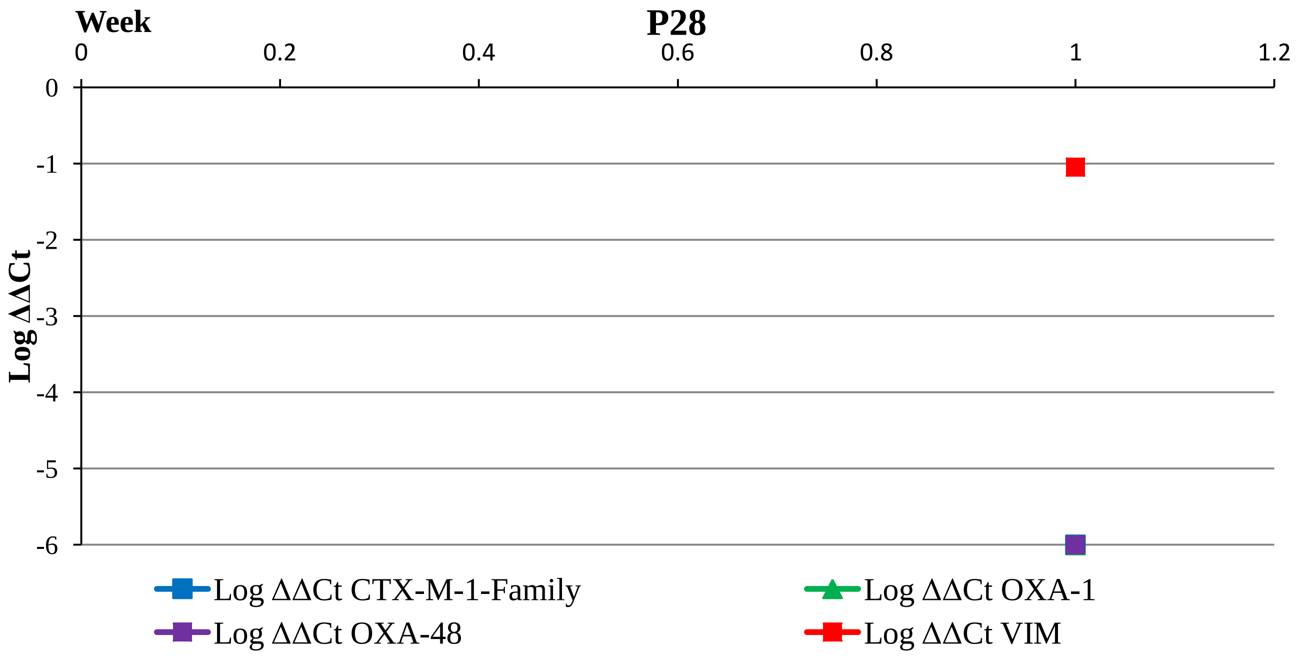
P28
Week
0
0.2
0.4
0.6
0.8
1
1.2
0
-1
-2
-3
-4
-5
-6
Log ΔΔCt
Log ΔΔCt CTX-M-1-Family
Log ΔΔCt OXA-1
Log ΔΔCt OXA-48
Log ΔΔCt VIM
